# Evaluation of Planned versus Unplanned Soft-Tissue Sarcoma Resection Using PROMIS Measures

**DOI:** 10.1155/2019/1342615

**Published:** 2019-03-05

**Authors:** Benjamin K. Wilke, Anna R. Cooper, Ashley K. Aratani, Mark T. Scarborough, C. Parker Gibbs, Andre Spiguel

**Affiliations:** Division of Orthopaedic Oncology, University of Florida, Gainesville, FL, USA

## Abstract

**Background:**

The Patient Reported Outcomes Measurement Information System (PROMIS) is a tool developed by the National Institutes of Health that allows comparisons across conditions or even the United States (U.S.) general population.

**Objectives:**

Our purpose was to compare PROMIS outcomes between patients who underwent a planned resection to those who underwent an initial unplanned excision of their sarcoma followed by a definitive oncologic resection. We then compared these groups to the U.S. general population.

**Methods:**

Eighty-five patients were included and were divided into those who underwent an initial planned resection (67) and unplanned excision (18). These patients were then further categorized based on the length of follow-up since their last surgery, either early (<12 months) or late (>12 months).

**Results:**

We evaluated seven PROMIS domains and found no differences between patients who underwent planned resection versus those who underwent an initial unplanned excision followed by a wide resection of the previous wound bed. When compared to the U.S. population, both cohorts demonstrated significantly improved scores in several emotional health domains.

**Conclusions:**

Patients who undergo an unplanned excision followed by a definitive oncologic procedure have similar PROMIS scores compared to patients who undergo an initial planned resection.

## 1. Introduction

The Patient Reported Outcomes Measurement Information System (PROMIS) is an outcome tool that was developed by the United States (U.S.) National Institutes of Health. It is a patient-reported tool that categorizes responses into health domains. These domains cover aspects of physical, mental, and social health. A unique and attractive feature of the PROMIS system is the ability to standardize responses. In converting individual patient responses to T-scores, a researcher can evaluate the health impact a rare entity such as a sarcoma has on a patient's life and compare this to a more common disease process, or even the U.S. general population [1]. PROMIS accomplishes this by converting scores from the United States general population to a T-score of 50, with a standard deviation of 10. In evaluating outcomes with the PROMIS tool, a lower score signifies less of the tested function; for example, if a patient scored 40 in the physical function domain, they would have a lower physical function level compared to the U.S. general population. Conversely, a score of 40 in the depression domain would indicate that the patient has a lower level of depression. The desired score is therefore dependent on the domain being tested [2].

The ability to compare across disease entities and to the U.S. general population allows physicians to more accurately explain the impact a certain diagnosis or treatment will have on a patient's life and allow the physician to explain this in a way that the patient is more apt to understand. Despite the advantages of the PROMIS system over previous outcome tools, such as the Musculoskeletal Tumor Society Scoring System (MSTS) or Toronto Extremity Salvage Score (TESS), which are oncology-specific and do not allow such broad comparisons, few orthopedic oncology studies have utilized the PROMIS system [3–5]. Our purpose in this study, therefore, was to compare patients who had undergone an unplanned excision of a nonmetastatic sarcoma and required a definitive oncologic resection to those who had undergone a planned initial resection. We attempted to answer the following questions: (1) Is there a difference in PROMIS scores between patients who underwent a planned versus unplanned resection of a nonmetastatic sarcoma? (2) Are these scores significantly different than the U.S. general population? In addition to answering these questions, we also provide normative values for future comparison studies.

## 2. Methods

We collected PROMIS data on all orthopedic oncology patient visits beginning September 1, 2016, as our standard of care. Prior to the visit with the physician, the patient was led to the clinic room, and a nurse loaded the questionnaire onto a computer for the patient to complete. The patient was given adequate time to complete the questionnaire before the physician entered the room. If the patient had difficulty completing the questionnaire, the nurse was available to assist.

**Figure 1:**
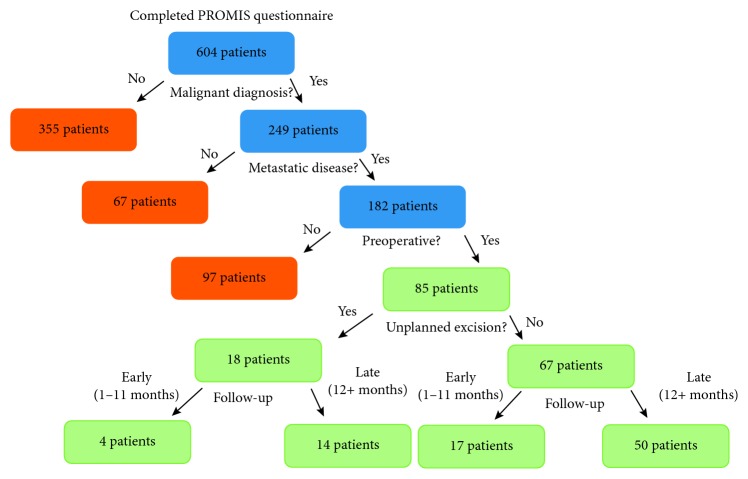
Flow chart of exclusion and inclusion criteria. Eligible patients are labeled in blue, excluded patients in orange, and patients included in the final analysis in green.

Following approval from our institutional review board (IRB), we queried this database from September 1, 2016, to December 31, 2016. Six hundred four patients had completed a PROMIS questionnaire during the study period. We excluded patients with benign disease, those with nononcologic diagnoses, metastatic lesions, and those who did not undergo the final surgery at our institution. We additionally excluded patients with osseous lesions. Eighty-five patients were included in the final analysis. We subdivided these patients into those who underwent a planned resection and those who underwent an unplanned excision and subsequently required an additional resection for definitive management. Finally, the cohorts were then divided into early, defined as less than 12 months from their last surgical procedure, and late, defined as greater than 12 months from their last surgical procedure (Figure 1).

We obtained demographic data and pathologic diagnoses from review of the patient's chart. Follow-up was determined based on the last surgical procedure rather than the initial resection to allow appropriate recovery time for patients who underwent multiple surgical procedures or wound complications. We used the latest PROMIS questionnaire in the analysis.

The PROMIS 43 Profile was used to collect data. This consists of short forms for seven health domains. The health domains include physical function, anxiety, depression, fatigue, sleep disturbance, ability to participate, and pain interference. Physical function measures patient perception of their physical function and ability to participate in activities of daily living. Anxiety, depression, fatigue, sleep disturbance, and pain interference evaluate the difficulties with each in their respective domains. Finally, ability to participate evaluates the patient's perception of their ability to participate in normal social activities [6]. Raw scores were converted to T-scores in order to allow for comparison with the United States general population.

### 2.1. Statistical Methods

The Profile 43 PROMIS questionnaire was completed by patients during routine clinic visits and stored in the electronic medical record. Incomplete entries were assessed per the PROMIS guidelines; briefly, if more than 50% of the modality entries were completed, then the raw score was calculated and adjusted for the number of missing entries. All raw scores were used to determine T-scores using the standard PROMIS T-score scales for adults. Mean T-scores were compared using the 2-tailed *t*-test where equal variance was not assumed. Significance was set at less than 0.05.

## 3. Results

Eight-five patients were included in the final analysis, including 43 males (51%) and 42 females (49%). Nineteen patients (22%) had tumors located in the upper extremity compared to 66 (78%) who had tumors located in the lower extremity. The average age was 63 years. We found no significant differences in tumor locations between groups. We also found no significant differences in the rates of limb salvage between the cohorts. We did observe a significant difference in the rate of adjuvant therapy between the cohorts, with a higher percentage of patients in the planned cohort receiving adjuvant treatment. Finally, there was no significant difference in postoperative complications between cohorts or average resection size (Table 1). The average resection size was based on the pathologic sample obtained during the definitive oncologic procedure. This included the tumor for the planned surgical resection and the wound bed for the unplanned excision cohorts.

**Figure 2:**
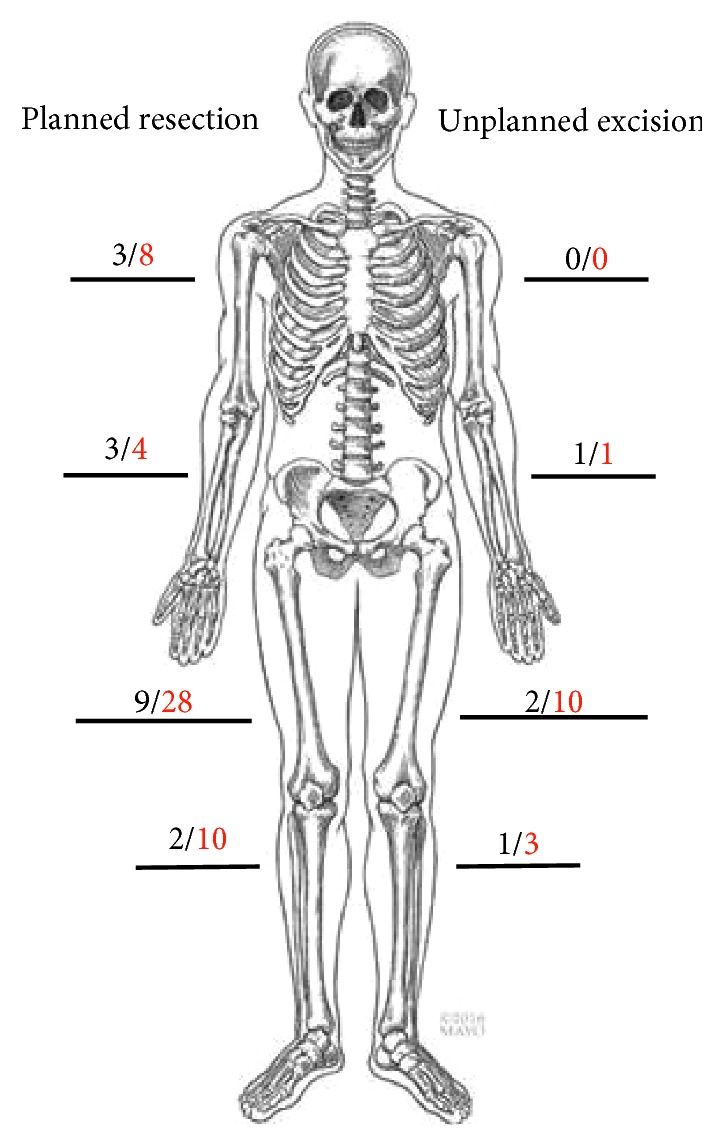
A diagram representing the locations of the tumor resections. Black represents early follow-up (<12 months), and red represents late follow-up (12 + months).

Patients were divided into those who had undergone an unplanned excision during their first surgical procedure and patients who had undergone a planned surgical resection. They were then further subdivided based on the acuity of the last surgical procedure, with those who had undergone a procedure within the last 12 months in the early follow-up group and those who were greater than one year from surgery in the late follow-up cohort.  Figure 2 shows the location of the tumor resections.  Table 2 lists the most common diagnoses.

In the planned resection group, nine patients (13%) required amputative procedures. Seven of these tumors were located in the lower extremity, and two were located in the upper extremity. In comparison, three patients (17%) in the unplanned excision group required an amputation. All three of these tumors were located in the lower extremity. There was no significant difference in the rate of amputations between the cohorts (*p*=0.728).

Adjuvant therapy, consisting of radiation therapy and/or chemotherapy, was given to 54 patients (81%) who underwent a planned resection. This is compared to ten patients (56%) in the unplanned excision cohort. There was a significant difference in the rate of adjuvant therapy received between cohorts (*p*=0.03). The majority of planned resection patients received preoperative radiation therapy compared to postoperative radiation in the unplanned excision cohort. The median time between the completion of adjuvant therapy and the survey was 10 months in the early unplanned group, 18 months in the late unplanned group, 0 months in the early planned resection group, and 29 months in the late planned group. Five patients in the early planned resection cohort were receiving postoperative chemotherapy during the completion of the survey, compared to one patient in the late planned cohort and no patients in the unplanned cohorts. The average PROMIS T-scores are seen in (Table 3).

The average PROMIS values based on the acuity of the last surgical procedure (early versus late) as well as the initial type of resection (planned versus unplanned) are shown in Table 4. We found a significant difference in depression scores based on the acuity of the surgical procedure, with scores decreasing (improving) in the later follow-up. We did not find a significant difference in the remaining PROMIS values based on the acuity of the surgical procedure. Additionally, we also did not find a significant difference in the PROMIS values based on the type of initial resection performed.

**Figure 3:**
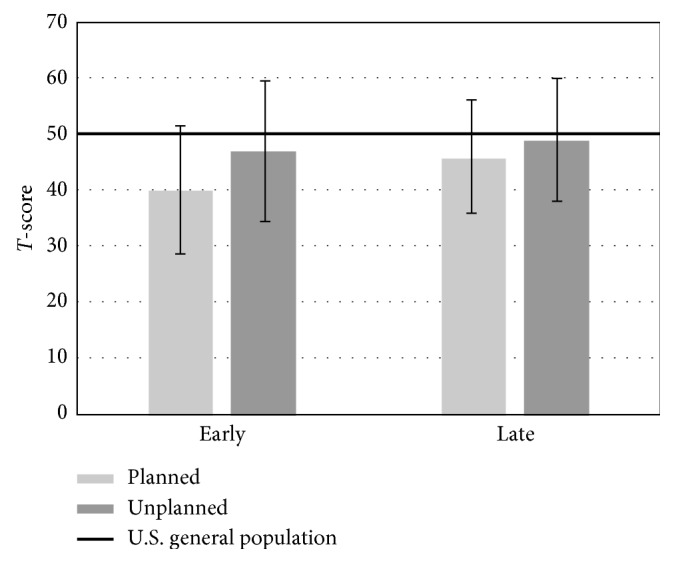
Average T-score values for the physical function domain based on acuity and type of surgical procedure. A significant difference was found between the late planned resection cohort and the U.S. general population (*p* ≤ 0.001).

**Figure 4:**
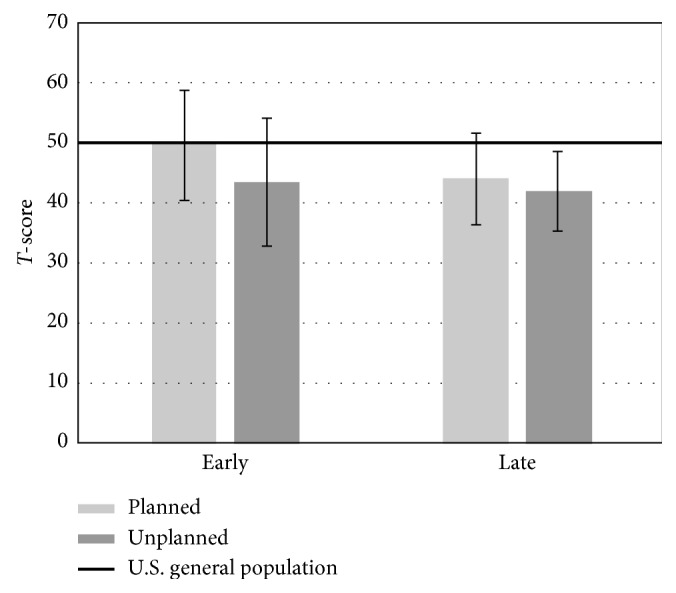
Average T-score values for the depression domain based on acuity and type of surgical procedure. A significant difference was found between both the late planned resection and unplanned excision cohorts and the U.S. general population (*p* ≤ 0.001 and *p*=0.001, respectively).

**Figure 5:**
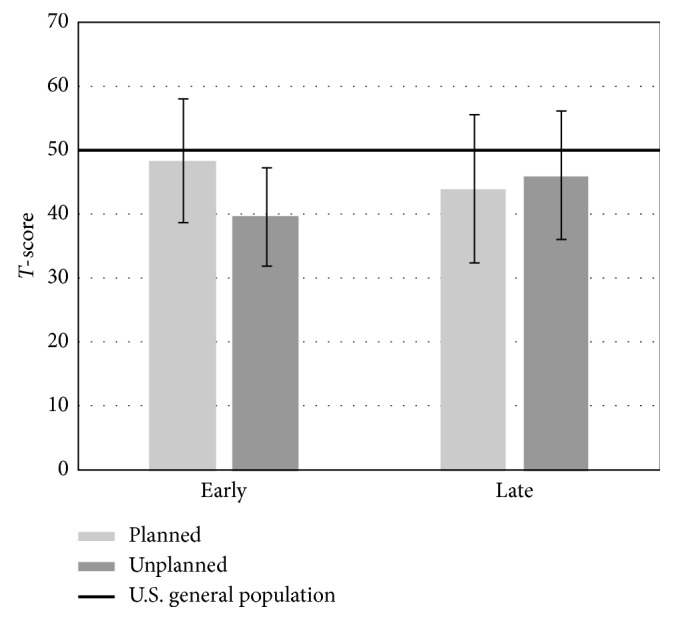
Average T-score values for the fatigue domain based on acuity and type of surgical procedure. A significant difference was found between both the late planned resection and unplanned excision cohorts and the U.S. general population (*p*=0.026 and *p*=0.027, respectively).

We then compared the patients in the late cohorts (12+months from the last surgical procedure) to the U.S. general population (Table 5). Several significant differences were found. We found a significantly lower physical function score in the planned resection cohort when compared to the U.S. general population (*p* ≤ 0.001). This was not reproduced in the unplanned excision group (*p*=0.708). We also found significantly lower depression and fatigue levels in both the planned and unplanned resection cohorts and significantly lower levels of sleep disturbance in the planned resection cohort compared to the U.S. general population. These values are represented graphically in Figures 3–5.

## 4. Discussion

Several studies have previously evaluated the impact an unplanned excision has on local recurrence and survival, but there is a paucity of data evaluating patient-reported functional outcomes in this population [7–11]. The aim of our study was therefore to compare results in patients who had undergone an unplanned excision and required a repeat resection to those patients who had undergone a planned initial resection. We attempted to answer whether there was a difference in PROMIS scores between these patient cohorts, as well as if these scores differed from the United States general population.

There are several unavoidable limitations in our study. As these patients were from a single institution and sarcomas are rare, our numbers are limited. Additionally, there is a wide variety in histologic diagnoses as well as tumor locations in our patient population. However, previous research has reported no significant impact on functional results based on anatomic location alone [12]. Future studies will need to independently verify our reported results.

A benefit of the PROMIS scoring system compared to previous systems is the ability to standardize the results. In doing this, we are able to compare our patients to a subset of the United States general population. When this comparison is made, several interesting differences are found. As one might expect, we noted a statistically lower score in the physical function domain in the planned resection cohort compared to the United States general population. This difference was not observed in the unplanned excision population. A reason for this discrepancy is likely due to the small numbers in our study. Alternatively, as Aria et al. suggests, this may also be due to less muscle resection in the unplanned cohort due to a more superficial location in these tumors, although we found no significant difference in the sizes of our resected specimens between groups [13].

Significant differences were also observed in our patients compared to the U.S. general population in several emotional health domains. Patients reported less difficulty with depression, fatigue, and sleep disturbance when compared to the U.S. general population. A potential explanation for the improvements in emotional health compared to the U.S. general population may be due to altered expectations once they are given a diagnosis of cancer and have undergone treatment for this. Previous studies have reported similar outcomes in cancer patients [14–16]. Future studies will need to verify this result.

When comparing PROMIS scores between cohorts we found no significant differences for physical function, emotional health, and social health domains based on whether patients underwent an unplanned excision prior to a definitive resection or if they underwent an initial planned resection. This finding is similar to studies that report no difference in functional results between unplanned excision and planned resection cohorts [13, 17, 18].

In addition to equivalent PROMIS scores between the unplanned excision and planned resection cohorts, we also noted a similar rate in ability to perform a limb salvage procedure between groups. In the planned resection cohort, a limb salvage procedure was performed in 87% of cases. This is compared to 83% in the unplanned excision cohort (*p*=0.728). Other studies have reported similar amputation rates between groups [13, 18, 19]. As before, Aria proposes that this finding may be due to the tumors in the unplanned cohort typically being smaller and located in a more superficial location compared to those that underwent an initial planned resection [13].

## 5. Conclusion

In conclusion, we found no significant difference in PROMIS scores between patients that underwent a planned resection for a nonmetastatic sarcoma compared to those who underwent an initial unplanned excision, followed by a definitive oncologic procedure. Both groups demonstrated improved emotional health scores compared to the U.S. general population. This result should not condone performing unplanned excisions but may be used to counsel patients who present following such a procedure.

## Figures and Tables

**Table 1:** Patient demographics.

	Unplanned excision (*N*=18)	Planned resection (*N*=67)	Total	*p* value
Sex				
Female	7 (39%)	35 (52%)	42 (49%)	0.505
Male	11 (61%)	32 (48%)	43 (51%)	
Upper extremity				
Yes	2 (11%)	17 (25%)	19 (22%)	0.2
No	16 (89%)	50 (75%)	66 (78%)	
Age (mean ± SD in years)	60 ± 18	64 ± 16	63 ± 16	0.415
Mean follow-up (including range, in months)	20 (1–80)	20 (1–272)	19 (1–272	0.216
Limb salvaged				
Yes	15 (83%)	58 (87%)	73 (86%)	0.728
No	3 (17%)	9 (13%)	12 (14%)	
Adjuvant treatment				
Yes	10 (56%)	54 (81%)	64 (75%)	0.03
No	8 (44%)	13 (19%)	21 (25%)	
Average resection size (cm)	12.6 (1.4–30)	10.6 (2.4–35)	11.0 (1.4–35)	0.309
Postoperative complications	4 (22%)	14 (22%)	18 (21%)	0.814
Median time from last adjuvant treatment to survey (months)				
<12 months	10 (3–17)	0 (0–2)	NA	0.407
>12 months	18 (11–67)	29 (0–86)	NA	0.951

**Table 2:** Most common pathologic diagnoses.

	Early		Late	
	Planned	Unplanned	Planned	Unplanned
Undifferentiated pleomorphic sarcoma	3	1	19	2
Liposarcoma	2	1	6	4
Myxofibrosarcoma	4	1	10	4
Synovial sarcoma	1	0	5	1
Spindle cell sarcoma	0	0	3	0
Extraskeletal chondrosarcoma	0	0	2	0
Leiomyosarcoma	2	0	2	2
Malignant peripheral nerve sheath tumor	2	0	0	0
Rhabdomyosarcoma	2	0	0	0
Epithelioid sarcoma	1	1	1	0
Others	0	0	2	1

**Table 3:** Mean values and standard deviation (SD) between planned resection and unplanned excision for the PROMIS domains.

Health domain	Procedure	Early		Late	
		Mean	SD	Mean	SD
Physical function T-score	Planned resection	40.0	11.5	45.5	10.1
	Unplanned excision	46.9	12.6	48.9	11.1

Anxiety T-score	Planned resection	52.4	9.6	47.3	8.9
	Unplanned excision	49.0	11.7	48.6	7.9

Depression T-score	Planned resection	49.6	9.2	43.9	7.7
	Unplanned excision	43.6	10.5	42.1	6.6

Fatigue T-score	Planned resection	48.3	9.7	46.7	11.6
	Unplanned excision	39.6	7.7	45.8	10.0

Sleep disturbance T-score	Planned resection	46.2	10.7	47.0	7.9
	Unplanned excision	46.6	5.7	44.4	10.4

Ability to participate T-score	Planned resection	45.9	13.2	52.0	11.5
	Unplanned excision	52.8	18.1	53.7	10.4

Pain interference T-score	Planned resection	54.6	12.6	50.6	10.7
	Unplanned excision	47.3	7.1	50.1	9.9

**Table 4:** Average values and standard deviation of PROMIS health domains based on the type of surgical procedure and acuity.

Dependent variable	Independent variable	Mean	SD	*p* value
Physical function T-score	*Acuity*			0.07
	Early	41.3	11.7	
	Late	46.2	10.3	
	*Surgical procedure*			0.136
	Planned resection	44.1	10.6	
	Unplanned excision	48.4	11.1	

Anxiety T-score	*Acuity*			0.069
	Early	51.7	9.8	
	Late	47.6	8.6	
	*Surgical procedure*			0.99
	Planned resection	48.6	9.3	
	Unplanned excision	48.7	8.4	

Depression T-score	*Acuity*			0.016
	Early	48.4	9.5	
	Late	43.5	7.4	
	*Surgical procedure*			0.186
	Planned resection	45.3	8.4	
	Unplanned excision	42.4	7.3	

Fatigue T-score	*Acuity*			0.97
	Early	46.6	9.8	
	Late	46.5	11.2	
	*Surgical procedure*			0.356
	Planned resection	47.1	11.7	
	Unplanned excision	44.4	9.7	

Sleep disturbance T-score	*Acuity*			0.951
	Early	46.3	9.8	
	Late	46.4	8.5	
	*Surgical procedure*			0.402
	Planned resection	46.8	8.6	
	Unplanned excision	44.9	9.5	

Ability to participate T-score	*Acuity*			0.087
	Early	47.2	14	
	Late	52.4	11.2	
	*Surgical procedure*			0.347
	Planned resection	50.5	12.1	
	Unplanned excision	53.5	11.9	

Pain interference T-score	*Acuity*			0.332
	Early	53.2	12	
	Late	50.5	10.5	
	*Surgical procedure*			0.458
	Planned resection	51.6	11.3	
	Unplanned excision	49.5	9.2	

**Table 5:** Average PROMIS values and standard deviations (SD) for patients at least 12 months following their surgical procedure.

Health domain	Procedure	Mean	SD	U.S. general population mean score	SD	*p* values
Physical function T-score	Planned resection	45.5	10.1	50	10	<0.001
	Unplanned excision	48.9	11.1			0.708

Anxiety T-score	Planned resection	47.3	8.9	50	10	0.172
	Unplanned excision	48.6	7.9			0.44.

Depression T-score	Planned resection	43.9	7.7	50	10	<0.001
	Unplanned excision	42.1	6.6			0.001

Fatigue T-score	Planned resection	46.7	11.6	50	10	0.026
	Unplanned excision	45.8	10.0			0.027

Sleep disturbance T-score	Planned resection	47.0	7.9	50	10	0.006
	Unplanned excision	44.4	10.4			0.055

Ability to participate T-score	Planned resection	52.0	11.5	50	10	0.465
	Unplanned excision	53.7	10.4			0.13

Pain interference T-score	Planned resection	50.6	10.7	50	10	0.264
	Unplanned excision	50.1	9.9			0.824

The U.S.s general population mean is set to 50 with a standard deviation of 10.

## Data Availability

The data used to support the findings of this study are available from the corresponding author upon request.
